# Half a Decade of Intravenous Ketamine Administration: Our Observation Results and Insights regarding its safety

**DOI:** 10.1192/j.eurpsy.2024.717

**Published:** 2024-08-27

**Authors:** P. Argitis, A. Karampas, M. Peyioti, T. Koukouras, S. Karavia, Z. Chaviaras

**Affiliations:** ^1^Psychiatric, General Hospital of Corfu, Corfu, Greece

## Abstract

**Introduction:**

Ketamine, originally an anesthetic, has emerged as a potent tool in the fight against treatment-resistant depression and suicide. Clinical trials have demonstrated its ability to induce remission of severe depressive symptoms, with effects that can extend over several weeks.Furthermore, research highlights Ketamine’s potential to rapidly reduce suicidal ideation. This suggests Ketamine’s role as an intervention in suicide prevention, especially when conventional treatments prove ineffective. While isolated cases report severe respiratory depression, primarily when combined with other medications, most incidents involve temporary apneic episodes following high-dose intravenous administration. Understanding Ketamine’s safety profile is vital for its clinical optimization and ensuring patient well-being during use

**Objectives:**

This presentation serves to describe, and evaluate our clinic’s safety protocol implemented for intravenous (IV) Ketamine infusions at the General Hospital of Corfu. Our primary goal is to rigorously assess the safety and tolerability of IV Ketamine in a clinical setting

**Methods:**

Patients must meet stringent criteria:

- Exclude those over 70.
- MMSE score above 25.
- Controlled blood pressure.
- No cardiac insufficiency, myocardial ischemia, or high intraocular/intracranial pressure.
- Absence of thyrotoxicosis, psychosis, or seizures.

Pre-infusion comprehensive evaluation:

- Includes ECG, blood biochemistry studies, and frequent blood pressure checks.
- Requires a 2-hour fast.

Ketamine infusion:

- IV Ketamine administered at 0.5mg/kg in 100ml N/S.
- Continuous monitoring of oxygen saturation (PO2) and cardiac rhythm.
- Blood pressure checks every 15 minutes.

Treatment typically involves 7 sessions over a span of a month, with an initial test dose of 0.25 mg/kg.

**Results:**

Ketamine infusions were administered to a total of 208 patients. The majority of participants experienced a slight increase in blood pressure, while there were no significant changes in cardiac rhythm. Additionally, almost all patients reported experiencing dizziness or headaches during the infusion. Notably, nearly half of the patients reported an alteration in taste perception as a side effect. It’s important to highlight that all observed side effects, spontaneously resolved within an hour after the conclusion of the infusion. However, in a small subset of cases (six instances), the side effects were severe enough to necessitate the premature termination of the ketamine infusion

**Conclusions:**

Although ketamine demonstrates a favorable safety profile with minimal major side effects when administered following our established safety protocol. However, we want to underscore the critical importance of vigilant patient monitoring during ketamine administration and the prompt addressing of any adverse effects. This proactive approach is paramount to ensure the safety and overall well-being of patients receiving ketamine treatment

**Disclosure of Interest:**

None Declared

